# Systematic review and updated network meta-analysis comparing open, laparoscopic, and robotic pancreaticoduodenectomy

**DOI:** 10.1007/s13304-020-00916-1

**Published:** 2020-12-14

**Authors:** Alberto Aiolfi, Francesca Lombardo, Gianluca Bonitta, Piergiorgio Danelli, Davide Bona

**Affiliations:** 1 Department of Biomedical Science for Health, Division of General Surgery, Istituto Clinico Sant’Ambrogio, University of Milan, Via Luigi Giuseppe Faravelli, 16, 20149 Milan, Italy; 2 Department of Biomedical and Clinical Sciences, “Luigi Sacco” Hospital, University of Milan, Milan, Italy

**Keywords:** Pancreaticoduodenectomy, Open pancreaticoduodenectomy, Laparoscopic pancreaticoduodenectomy, Robotic pancreaticoduodenectomy, Network meta-analysis

## Abstract

The treatment of periampullary and pancreatic head neoplasms is evolving. While minimally invasive Pancreaticoduodenectomy (PD) has gained worldwide interest, there has been a debate on its related outcomes. The purpose of this paper was to provide an updated evidence comparing short-term surgical and oncologic outcomes within Open Pancreaticoduodenectomy (OpenPD), Laparoscopic Pancreaticoduodenectomy (LapPD), and Robotic Pancreaticoduodenectomy (RobPD). MEDLINE, Web of Science, PubMed, Cochrane Central Library, and ClinicalTrials.gov were referred for systematic search. A Bayesian network meta-analysis was executed. Forty-one articles (56,440 patients) were included; 48,382 (85.7%) underwent OpenPD, 5570 (9.8%) LapPD, and 2488 (4.5%) RobPD. Compared to OpenPD, LapPD and RobPD had similar postoperative mortality [Risk Ratio (RR) = 1.26; 95%CrI 0.91–1.61 and RR = 0.78; 95%CrI 0.54–1.12)], clinically relevant (grade B/C) postoperative pancreatic fistula (POPF) (RR = 1.12; 95%CrI 0.82–1.43 and RR = 0.87; 95%CrI 0.64–1.14, respectively), and severe (Clavien-Dindo ≥ 3) postoperative complications (RR = 1.03; 95%CrI 0.80–1.46 and RR = 0.93; 95%CrI 0.65–1.14, respectively). Compared to OpenPD, both LapPD and RobPD had significantly reduced hospital length-of-stay, estimated blood loss, infectious, pulmonary, overall complications, postoperative bleeding, and hospital readmission. No differences were found in the number of retrieved lymph nodes and R0. OpenPD, LapPD, and RobPD seem to be comparable across clinically relevant POPF, severe complications, postoperative mortality, retrieved lymphnodes, and R0. LapPD and RobPD appears to be safer in terms of infectious, pulmonary, and overall complications with reduced hospital readmission We advocate surgeons to choose their preferred surgical approach according to their expertise, however, the adoption of minimally invasive techniques may possibly improve patients’ outcomes.

**Electronic supplementary material:**

The online version of this article (10.1007/s13304-020-00916-1) contains supplementary material, which is available to authorized users.

## Introduction

Pancreaticoduodenectomy (PD) is a technically demanding surgical procedure that can provide cure or improved survival in patients with periampullary and pancreatic head diseases [1]. Indications for resection have increased because of the improvements in neoadjuvant treatment and surgical technique [2, 3]. There has been a growing interest towards minimally invasive techniques since the description of the first Laparoscopic Pancreaticoduodenectomy (LapPD) in 1994 [4]. Since then, LapPD has gained increasing acceptance with studies demonstrating its feasibility, safety and promising results [5]. Its reputation, however, was limited by the criticism regarding the protracted learning curve, potential for higher morbidity and the need for a high hospital volume to reach outcomes comparable to the open technique [6, 7]. The growth of innovative robotic platforms has later opened new perspectives since the first Robotic-assisted Pancreaticoduodenectomy (RobPD) [8]. The better ergonomics, high-definition 3-D visualization, and increased motion allowed by the instruments with multiple degrees of freedom have contributed to the progressive worldwide spread of RobPD [9].

Previous studies have showed that LapPD and RobPD seem to be equally safe and were associated with reduced morbidity, shorter hospital length of stay, and non-inferior oncologic outcomes when compared to OpenPD [9–14]. The purpose of this network meta-analysis is to provide an updated evidence comparing short-term surgical and oncologic outcomes within pure OpenPD, LapPD, and RobPD.

## Materials and methods

The systematic review was completed using the preferred reporting items for systematic reviews and network Meta-analyses guidelines (PRISMA-NMA) [15]. Approval from the local institutional review board was not necessary.

MEDLINE, Web of Science, PubMed, Cochrane Central Library and ClinicalTrials.gov were utilized for systematic search [16]. Articles published up to November 30, 2019 were screened. We searched for articles using the following search strategy: (“open pancreaticoduodenectomy” [tiab], OR “open pancreatoduodenectomy” [tiab]) AND (“laparoscopic pancreaticoduodenectomy” [tiab], OR “laparoscopic pancreatoduodenectomy” [tiab]); (“laparoscopic pancreaticoduodenectomy” [tiab], OR “laparoscopic pancreatoduodenectomy” [tiab]) AND (“robotic pancreaticoduodenectomy” [tiab], OR “robotic pancreatoduodenectomy” [tiab]); (“open pancreaticoduodenectomy” [tiab], OR “open pancreatoduodenectomy” [tiab]) AND (“robotic pancreaticoduodenectomy” [tiab], OR “robotic pancreatoduodenectomy” [tiab]). Titles and abstracts were assessed, inspected and references were screened. The PROSPERO study protocol registration number is CRD42020170952.

### Eligibility criteria

Inclusion criteria includes (a) articles comparing surgical outcomes for either OpenPD, LapPD or RobPD in the setting of malignant, borderline or benign disease; (b) English-written studies; (c) articles with the longest follow-up or the largest sample size when two or more papers were published by the same institution, study group or used the same data-set; (d) studies released after the year 2003. Exclusion criteria are (a) non-English written articles; (b) studies without clear methodology and surgical technique; (c) articles reporting hybrid techniques (i.e., hand-assisted laparoscopic or robotic resections, combined laparoscopic-robotic approaches out of the standard); (d) studies with less than 20 patients per-arm comparison.

### Data extraction

Retrieved records were authors, nation, year of publication, study design, patients’ number, demographics, American Society of Anesthesiologists (ASA) score, surgical approach, postoperative surgical and oncologic outcomes. Three investigators (AA, FL, GB) individually extracted data from eligible articles and a fourth author (DB) clarified disagreements.

### Definitions

PD was defined as any method of surgical removal of the pancreatic head, duodenum, and distal common bile duct. RobPD was defined as the use of robotic technique for PD, including resection and reconstruction without laparoscopic or hand-assisted techniques, nevertheless, this did include the use of laparoscopic ports by a surgical assistant which is considered as the standard practice. LapPD was defined as the complete use of a laparoscopic technique for PD, including resection and reconstruction without robotic or hand-assisted techniques.

Postoperative mortality was defined as either in-hospital or within 90-day mortality after PD. Postoperative Pancreatic Fistula (POPF) was classified in accordance with the International Study Group of Pancreatic Surgery (ISGPS); grade B/C were considered clinically relevant [17]. Postoperative complications were recorded according to the Clavien-Dindo classification; grade ≥ 3 were considered as severe postoperative complications [18]. Delayed Gastric Empting (DGE) was defined according to the ISGPS; grade B/C were considered as clinically relevant [17].

### Quality evaluation

The quality of observational studies was assessed with the Risk of Bias In Non-Randomized Studies (ROBINS-I) instrument [19]. Confounding, selection, classification, intervention, missing data, outcomes measurement and reporting bias were considered. Each domain was estimated with “yes”, “probably yes”, “probably no” or “no” and studies were categorized as having low, moderate, serious, or critical risk of bias. The Cochrane risk of bias was adopted to appraise the quality of Randomized Controlled Trials (RCTs) and were graded as low risk (green circle), high risk (red circle), or unclear risk (yellow circle) of bias [20].

### Outcomes

Primary outcomes were postoperative mortality, grade B/C POPF, severe postoperative complications (Clavien-Dindo ≥ 3). Secondary outcomes were grade B/C DGE, Surgical Site Infection (SSI), pulmonary complications, bile leak, overall complications, estimated blood loss (ml), operative time (minutes), conversion to open, reoperation, hospital length of stay (HLOS) (days), postoperative bleeding, hospital readmission, R0, harvested lymphnodes (*n*), and costs (in $).

### Statistical analysis

A systematic review and a comprehensive Bayesian network analysis were executed [21–23]. Risk Ratio (RR) and estimated mean difference (md) were used as pooled effect size measure for binary and continuous outcomes. For the between-study variability (*τ*) we used an informative half-normal prior with zero mean and scale 0.5 [24]. To assess local inconsistencies, the node‐splitting method and prior distribution sensitivity analysis were measured [25, 26]. Heterogeneity (*I*^2^) was defined as low (< 25%), moderate (25–75%), or high (> 75%) [27]. The inference was performed using mean and 95% Credible Intervals (CrI) and was considered significant when it encompasses the null hypothesis value. The transitivity assumption (i.e., studies comparing different sets of interventions needed to be sufficiently similar) was considered to provide valid indirect inferences. To assess transitivity, we generated descriptive statistics and compare the distributions of baseline characteristics across studies and treatment comparisons. The accuracy of the inference was assessed by convergence of MCMC algorithm [28]. The treatment ranking probability was estimated with the cumulative ranking curve (SUCRA). The network geometry was appraised and the confidence of outcomes estimates was assessed with Confidence in Network Meta‐Analysis (CINeMA) instrument. Jags and R-Cran were used for statistical analyses [29].

## Results

### Systematic review

**Fig. 1:**
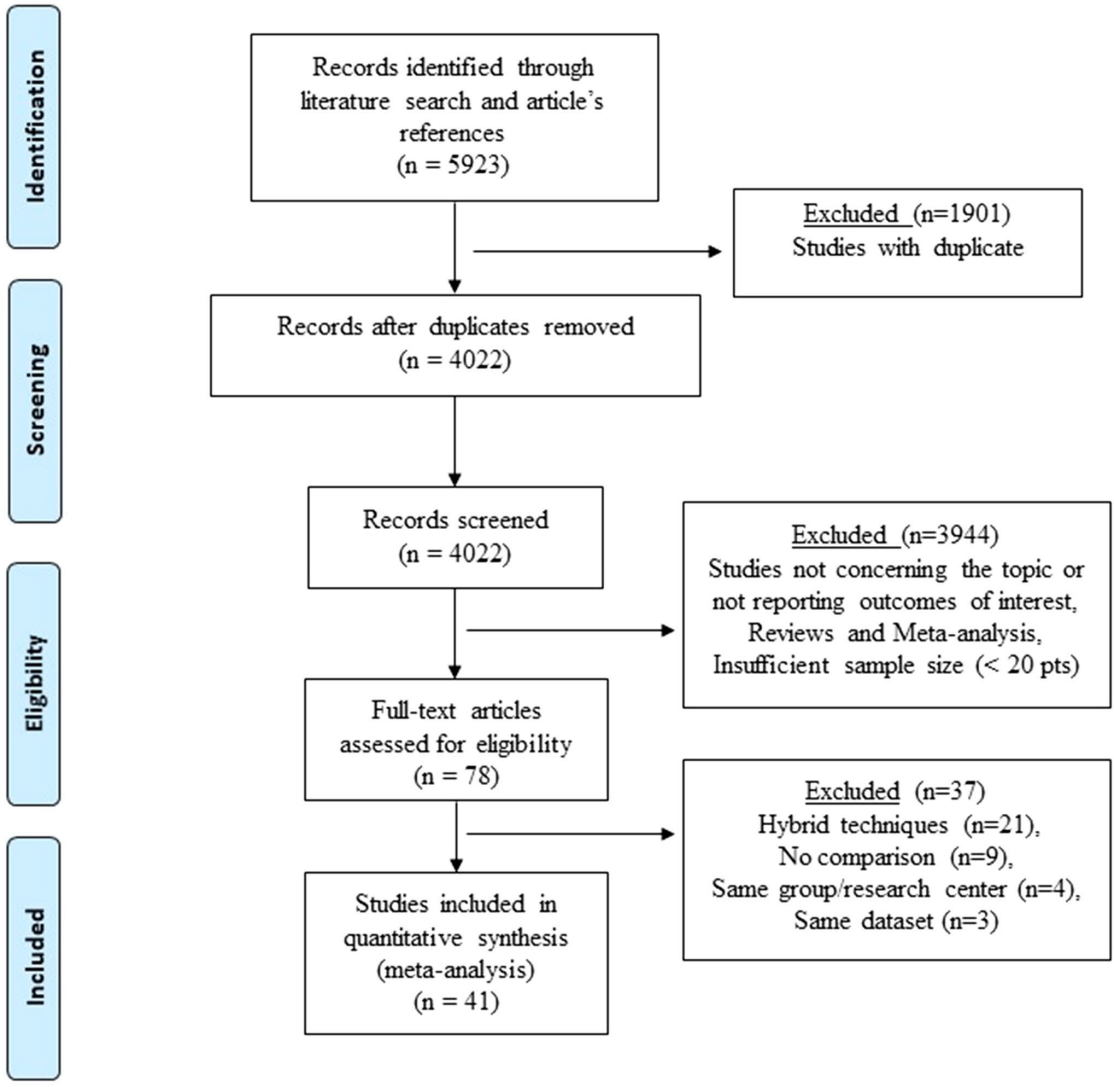
The Preferred Reporting Items for Systematic Reviews and network meta-analyses checklist (PRISMA-NMA) diagram

Five thousand nine hundred and twenty-three titles were found using the described criteria. After removing duplicates, 4022 publications were revised with 41 studies fulfilling the inclusion criteria (Fig. 1). Of the included studies, three were RCTs. None of the studies received a low risk of bias on all assessed items. Because the lack of patients and/or outcomes assessors blinding, all trials were graded as having high/unclear risk of performance and detection bias. Because the individual surgeon experience was not precisely indicated, other bias was defined as high (Supplementary Fig. 1). Thirty-eight articles were observational non-randomized studies. According to the ROBINS-I tool, 21 studies were categorized as having moderate risk of bias while 17 were categorized as having severe risk of bias. Outcomes might have been influenced by confounding and selection bias because inclusion/exclusion criteria and patient treatment allocation were heterogeneous among studies (Supplementary Table 1). The assessments of confidence in the estimates using CINeMA showed low to very low confidence, essentially due to study limitation, imprecision, and heterogeneity.

Patient demographics and preoperative characteristics are summarized in Table 1. Overall, 56,440 patients were included in the quantitative analysis, out of which 48,382 (85.7%) underwent OpenPD, 5570 (9.8%) LapPD, and 2488 (4.5%) RobPD. The median age (range) was 61.5 years (18–89). Gender was specified in 45,521 patients: 23,054 patients (50.6%) were male and 22,467 (49.4%) were females. BMI was defined in 26,253 patients; the median (range) BMI was 25.2 (15–49.6). ASA score was reported in 20,812 patients (20 studies) with 10,408 (50.1%) being classified as having ASA ≥ 3. Overall, 92.5% of patients underwent surgery for malignancy, 55.9% for pancreatic ductal adenocarcinoma, and 7.5% for borderline or benign tumor. Tumor size was defined in 21 studies and ranged from 0.1 to 14 cm. Neoadjuvant therapy was reported in 25 studies and consisted in any preoperative chemotherapy, radiation therapy or both. Cost analysis was reported in six studies.

**Table 1:** Demographic and clinical characteristics of patients according to different treatment

Author, year, country	Study period	Study design	Surgical procedure	No. patients	Age (yrs)	Gender M/F	BMI (kg/m^2^)	ASA ≥ 3 (%)	PDAC (%)—Malignancy (%)	Tumor Size (cm)	Neoadjuvant (%)	Vascular involvement (%)
Asbun et al. USA [30]	2005–2011	Ret	OpenPD	215	67.3 ± 11.5	95/120	26.6 ± 5.1	81.8	46.5–84	3.1 ± 1.5	4.3	22.3
			LapPD	53	62.9 ± 14.1	29/24	27.4 ± 7.1	75.4	41.5–94	2.7 ± 1.6	7.7	9.4
Croome et al. USA [31]	2007–2013	Ret	OpenPD	214	65.4 ± 10.9	131/83	27.2 ± 5.3	nr	100–100	3.3 ± 1.3	14	40
			LapPD	108	66.6 ± 9.6	51/57	27.4 ± 5.4		100–100	3.3 ± 1	11.1	20
Dokmak et al. France [32]	2011–2014	Ret	OpenPD	46	63 (47–81)	28/18	26.4 (19–42)	nr	30–78	nr	0	0
			LapPD	46	60 (27–85)	26/20	22.6 (17–30)		32–78		0	0
Sharpe USA [33]	2010–2011	Ret, database	OpenPD	4037	65.6 ± 10	nr	nr	nr	100–100	3.3 ± 2.4	20	nr
			LapPD	384	66.1 ± 10.8				100–100	3.2 ± 1.3	18	
Speicher et al. USA [34]	2010–2013	Ret	OpenPD	84	64 (58–72)	36/48	25 (22–29)	nr	nr–73	3 (2–4)	31	0
			LapPD	25	61 (57–69)	16/9	24 (24–29)		nr–83	2 (1–4)	36	0
Tee et al. USA [35]	2007–2014	Ret	OpenPD	225	76.4 ± 4.5	140/85	26.8 ± 4.3	70	54–85.3	nr	4.9	17.3
			LapPD	113	76.5 ± 4.3	51/62	26.9 ± 4.7	73.4	57–66.4		4.4	15.9
Tran et al. USA [36]	2000–2010	Ret, database	OpenPD	14,893	65 (56–73)	7701/7192	nr	nr	nr	nr	nr	nr
			LapPD	681	67 (58–73)	377/304						
Senthilnathan et al. India [37]	2006–2011	Ret	OpenPD	118	56 ± 10	59/59	28.1	nr	47.4–100	3.1	0	0
			LapPD	45	54 ± 11	17/28	27.6		47–100	2.8	0	0
Tan China [38]	2009–2014	Ret	OpenPD	30	60 ± 10	23/7	nr	20	77–87	nr	nr	nr
			LapPD	30	59.3 ± 9	18/12		16.7	83–90			
Song et al. South Corea [39]	2007–2012	Ret	OpenPD	198	54.9 ± 12	100/98	23.9 ± 2.9	nr	37.4–100	3.4 ± 2.1	0	0
			LapPD	97	48.6 ± 14	49/49	22.7 ± 2.8		43.3–100	3.1 ± 1.4	0	0
Kantor USA [40]	2010–2013	Ret, database	OpenPD	7385	65.7 ± 10	nr	nr	nr	100–100	nr	19.9	nr
			LapPD	828	65.9 ± 10		22.7 ± 2.8		100–100		19.3	
Delitto et al. USA [41]	2010–2014	Ret	OpenPD	50	68.6 (1.4)	28/22	25.5 (0.7)	nr	44–100	3.1 (0.2)	6	0
			LapPD	52	65.3 (1.7)	34/18	26.3 (0.8)		54–100	2.5 (0.1)	6	0
Stauffer et al. USA [42]	1995–2014	Ret	OpenPD	193	69 (33–87)	96/97	25.6 (15–46)	79.2 72.4	100–100	3.5 (0.3–14)	8.8	31
			LapPD	58	70 (40–84)	32/26	25.9 (17–49)		100–100	2.5 (0.3–10)	6.9	34
Conrad et al. USA [43]	2000–2010	Ret	OpenPD	25	66 (43–76)	18/7	23.9 (20–31)	nr	76–100	nr	8	16
			LapPD	40	65 (45–83)	26/14	24.1 (14.9–34)		67.5–100		7.5	7.5
Chopinet Ftance [44]	2002–2014	Ret, PS	OpenPD	65	62 (19–84)	nr	23 (17–38)	nr	59–82	nr	nr	18
			LapPD	65	62 (31–83)		23 (15–35)		38–78			9
Palanivelu India [45]	2013–2015	RCT	OpenPD	32	58.6 ± 2	22/10	22.4 ± 0.6	9.3	25–100	3.6 ± 1.9	nr	9.4
			LapPD	32	57.8 ± 2	18/14	24.9 ± 0.7	6.2	9.5–100	3.3 ± 0.7		3
Chapman USA [46]	2010–2013	Ret, database	OpenPD	1520	79.5 (3.4)	721/799	nr	nr	100–100	nr	13.6	nr
			LapPD	248	79.6 (3.5)	132/116			100–100		12.3	
Poves Spain [47]	2013–2017	RCT	OpenPD	29	70 (36–83)	20/9	26 (17–43)	51.7	72–87	2.9 (1.2–7.5)	0	13.8
			LapPD	32	69 (34–86)	13/19	24 (16–33)	40.6	52–91	2.4 (0.9–7)	0	12.5
Chen China [48]	2013–2017	Ret	OpenPD	55	66 ± 15	34/21	22.7 ± 3.3	nr	54.5–100	3 ± 1.8	nr	0
			LapPD	47	63 ± 12	26/21	24 ± 3		62–100	2.5 ± 1.5		0
Kuesters Germany [49]	2001–2016	Ret	OpenPD	278	68	137/141	24 (16–46)	7.4 2.8	100–100	2.7 (0.3–13)	nr	43
			LapPD	62	71	31/31	24 (15–39)		100–100	2.8 (0.1–7.5)		40
Meng et al. China [50]	2010–2015	Ret	OpenPD	58	60.3 ± 8.6	34/24	22.9 ± 2.3	41.4	60.4–100	nr	nr	nr
			LapPD	58	59.9 ± 9.1	32/26	22.2 ± 2.9	51.7	58.6–100			
Van Hilst Netherlands [51]	2016–2017	RCT	OpenPD	49	66 (61–73)	25/24	26 ± 4	32.6	31–100	2.6 ± 1.2	0	4
			LapPD	50	67 (59–76)	30/30	25 ± 3	26	28–100	2.6 ± 1.4	0	10
Buchs USA [52]	2002–2010	Ret	OpenPD	39	56 ± 15.8	14/25	24.8 ± 4.7	nr	nr − 70	nr	nr	4.6
			RobPD	44	60 ± 14.5	22/22	27.7 ± 5.4		nr − 75			0
Lai China [53]	2000–2012	Ret	OpenPD	67	62.1 ± 11–2	38/29	nr	0	36–80	2.9 ± 2.3	nr	0
			RobPD	20	66.4 ± 11.9	12/8		0	35–75	2.1 ± 0.7		0
Baker USA [54]	2012–2013	Ret	OpenPD	49	63 (26–86)	31/18	27.7 (16.2–38.2)	81.6	61.2–81	3.1 (0–14.9)	12.8	14.3
			RobPD	22	63 (38–82)	13/9	25.5 (18.2–35.1)	68.2	68.2–82	2.7 (1.5–6)	10	13.6
Chen USA [55]	2010–2013	Pros	OpenPD	120	53.8 ± 14.3	65/55	22.6 ± 3.4	1.6	31.7–65	2.6 ± 1.3	0	nr
			RobPD	60	53.6 ± 13.5	34/26	23.2 ± 2.7	1.7	31.7–63.3	2.6 ± 1.5	0	
Girgis USA [56]	2011–2015	Ret	OpenPD	75	64.1	46/29	34,6	nr	49–83	nr	39	21
			RobPD	70	65.7	37/33	34		41–78.7		27	10
Boggi Italy [57]	2008–2014	Pros	OpenPD	36	64 (56–74)	19/17	23.4 (22–24.8)	44.4	30.5–83.3	nr	nr	11
			RobPD	83	62 (50–71)	37/46	23.8 (22–24.3)	32.5	19.3–85.6			8.4
McMillan et al. USA [58]	2013–2015	Pros	OpenPD	152	nr	nr	nr	nr	44.9–nr	nr	nr	nr
			RobPD	152					50.2–nr			
Zureikat USA [59]	2011–2015	Ret	OpenPD	817	65 (15–93)	427/390	26.1 (14.7–85.5)	nr	55–81	nr	nr	nr
			RobPD	211	67 (15-86)	117/94	27.5(18.1-47.6)		33 - 76			
Varley USA [60]	2011–2016	Ret	OpenPD	149	67 ± 10.5	79/70	26.7 ± 5.6	85.9	76.5–nr	nr	nr	nr
			RobPD	133	66.3 ± 10.6	64/69	27.5 ± 6.1	88.7	67–nr			
Kauffmann et al. Italy [61]	2014–2017	Ret, PS	OpenPD	26	70.8 ± 5.5	13/13	24.1 ± 3.1	nr	nr − 100	nr	0	21
			RobPD	24	65.8 ± 4.6	10; 14	23.1 ± 3.2		nr − 100		0	25
Napoli et al. Italy [62]	2007–2014	Ret, PS	OpenPD	227	67.3 ± 2.5	125/102	24.8 ± 0.2	65.6	42–76	nr	nr	36.6
			RobPD	82	61.5 ± 3.1	36/46	23.5 ± 0.4	41.4	28.1–64.6			8.6
Wang et al. Taiwan [63]	2012–2017	Ret, PS	OpenPD	87	nr	44/43	nr	nr	34.5–90	nr	nr	nr
		Ret, PS	RobPD	87		42/45			33.3–83			
Cai USA [64]	2011–2018	Ret	OpenPD	405	67.5 ± 10.7	211/194	27.2 ± 5.9	nr	57 − nr	nr	47	23
			RobPD	460	66.5 ± 11	253/207	27.8 ± 5.8		49 − nr		40.3	14.6
Marino et al. Italy [65]	2014–2016	Pros	OpenPD	35	62.3 (45–73)	nr	23.5 (18.8–28)	22.8	37–97	2.2 (1.2–3.5)	11.4	nr
			RobPD	35	60.4 (43–72)		23.8 (19.4–31)	20	46–94	2.3 (1.6–3.4)	17.1	
Liu China [66]	2015–2016	Ret	LapPD	25	60.54 ± 18.25	12/13	nr	0	20–92	1.9 ± 1.29	nr	0
			RobPD	27	57.16 ± 8.56	14/13		0	26–89	2.24 ± 1.6		0
Nassour USA [67]	2014–2015	Ret, NSQUIP	LapPD	235	63.4 ± 11.6	129/106	27.6 ± 6.6	75.7	nr–80	nr	25.6	23.4
			RobPD	193	63.5 ± 11.9	101/92	27.8 ± 5.3	76.6	nr–80		24.4	12.4
Nassour USA [68]	2010–2013	Ret, NSQUIP	LapPD	1458	66.3 (67)	756/702	nr	nr	89.1–100	nr	10	nr
			RobPD	165	66.5 (67)	81/84			90.1–100		10	
Zimmerman USA [69]	2014–2015	Ret, NSQUIP	OpenPD	6336	65 (57–72)	3411/2925	26.5 (23.2–32)	6.7	nr − 77.6	nr	22.4	17.9
			LapPD	280	64 (57–72)	159/121	26.9 (23.5–31)	2.5	nr − 78.3		24.1	21.5
			RobPD	211	66 (68–72)	109/102	27.3 (23.8–301)	5.7	nr − 57.6		22.3	11.7
Xourafas et al. USA [70]	2014–2016	Ret, NSQUIP	OpenPD	9963	65 (18–89)	5359/4604	27.2 (15–69)	77	56–81	nr	24	4
			LapPD	418	63 (19–87)	233/185	27.6 (16–67)	74.4	52–76		23	4
			RobPD	409	64 (18–88)	216/193	27.5 (19–51)	78.7	54–76		24	2

*OpenPD*: Open Pancreaticoduodenectomy, *LapPD*: Laparoscopic Pancreaticoduodenectomy, and *RobPD*: Robotic Pancreaticoduodenectomy. *BMI*: Body Mass Index. *ASA*: American Society of Anesthesiologists Classification. *PDAC*: Pancreatic ductal adenocarcinoma. *Ret*: Retrospective; *PS* Propensity Score matching; *Pros*: Prospective; *RCT*: Randomized Controlled Trial. Data are reported as mean ± standard deviation, median (range), and number. *nr* not reported

Neoadjuvant therapy consisted in any preoperative chemotherapy, radiation therapy or both

### Meta-analysis

#### Postoperative mortality

**Fig. 2:**
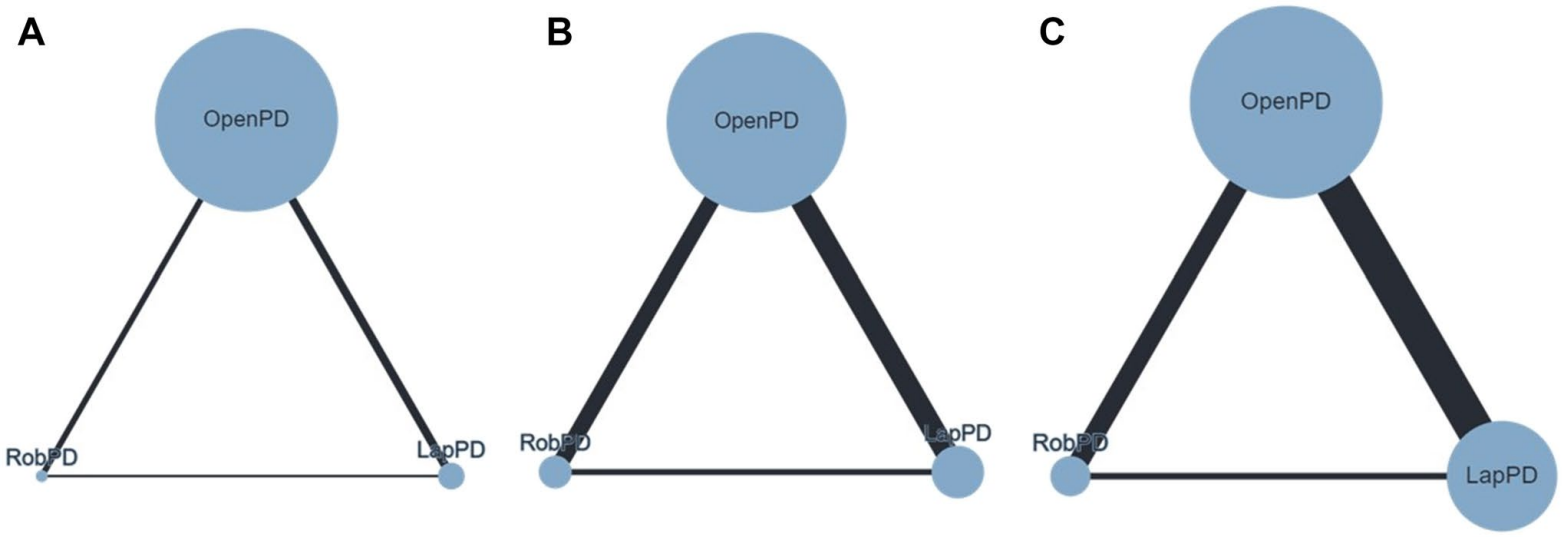
Network geometry for studies reporting: (**a**) Postoperative mortality, (**b**) grade B/C POPF, (**c**) Clavien-Dindo 3/4. The nodes reflect the surgical approaches (OpenPD, LapPD, and RobPD) while the connecting edge reflect the treatment comparison. Nodes size reflects the sample size while edges width reflects the number of studies for a specific pairwise comparison

Thirty-eight studies (54,179 patients) reported this outcome (Fig. 2a). No significant differences were found comparing LapPD vs. OpenPD (RR = 1.26; 95%CrI 0.91–1.61) and RobPD vs. OpenPD (RR = 0.78; 95%CrI 0.54–1.12). The global heterogeneity was low (*I*^2^ = 18.2%; 95%CrI 10.1–29.2%). The SUCRA ranking was 94% for LapPD, 52% for OpenPD, and 4% for RobPD (Supplementary Fig 2A). The node splitting analysis does not show evidence of local inconsistency and the sensitivity analysis yields closer results for LapPD vs. OpenPD (RR = 1.31; 95%CrI 0.95–2.03) and RobPD vs. OpenPD (RR = 0.72; 95%CrI 0.45–1.13).

#### Grade B/C POPF

Thirty-six studies (50,974 patients) reported this outcome (Fig. 2b). No significant differences were noticed for LapPD vs. OpenPD (RR = 1.12; 95%CrI 0.82–1.43) and RobPD vs. OpenPD (RR = 0.87; 95%CrI 0.64–1.14). The global heterogeneity was low (*I*^2^ = 21.2%; 95%CrI 13.4–28.6%). The SUCRA ranking was 78% for LapPD, 58% for OpenPD, and 13% for RobPD (Supplementary Fig. 2B). The node splitting analysis does not show evidence of local inconsistency and the sensitivity analysis produces closer results for LapPD vs. OpenPD (RR = 1.14; 95%CrI 0.84–1.47) and RobPD vs. OpenPD (RR = 0.85; 95%CrI 0.61–1.13).

#### Severe postoperative complication (Clavien-Dindo ≥ 3)

Twenty-eight studies (48,921 patients) reported this outcome (Fig. 2c). No significant differences were found comparing LapPD vs. OpenPD (RR = 1.03; 95%CrI 0.80–1.46) and RobPD vs. OpenPD (RR = 0.93; 95%CrI 0.65–1.14). The global heterogeneity was low (*I*^2^ = 24.2%; 95%CrI 15.6–31.8%). The SUCRA ranking was 68% for LapPD, 53% for OpenPD, and 29% for RobPD (Supplementary Fig. 2C). The node splitting analysis does not show evidence of local inconsistency and the sensitivity analysis shows similar results for LapPD vs. OpenPD (RR = 1.15; 95%CrI 0.89–1.52) and RobPD vs. OpenPD (RR = 0.86; 95%CrI 0.71–1.29).

#### Secondary outcomes

The pooled analysis shows a significantly reduced RR comparing LapPD vs. OpenPD and RobPD vs. OpenPD in terms of surgical site infection (20 studies) (RR = 0.71; 95% CrI 0.59–0.83 and RR = 0.68; 95%CrI 0.54–0.81, respectively), pulmonary complications (18 studies) (RR = 0.81; 95%CrI 0.70–0.94 and RR = 0.73; 95%CrI 0.64–0.86, respectively), overall complications (25 studies) (RR = 0.81; 95% CrI 0.74–0.93 and RR = 0.79; 95%CrI 0.72–0.91, respectively), and hospital readmission (17 studies) (RR = 0.81; 95% CrI 0.70–0.94 and RR = 0.73; 95%CrI 0.64–0.86, respectively).

Compared to OpenPD, both LapPD and RobPD showed a significantly reduced HLOS (35 studies) (md − 1.9; 95%CrI − 3.01, − 0.77 and -2.23; 95%CrI − 3.6, − 0.99, respectively), estimated intraoperative blood loss (29 studies) (md –148.5; 95%CrI − 156.5, − 140.5 and − 158.5; 95%CrI − 169.2, − 147.9, respectively), and postoperative bleeding (23 studies) (RR = 0.64; 95%CrI 0.48–0.81 and RR = 0.67; 95%CrI 0.42–0.82, respectively). Operative time was significantly longer when comparing LapPD vs. OpenPD and RobPD vs. OpenPD (md 60.9; 95% CrI 53.9–67.98 and md = 33.1; 95%CrI 24.02–42.3, respectively) (32 studies). RobPD was associated with significantly reduced risk of conversion to open compared to LapPD (RR = 0.71; 95% CrI 0.59–0.83; *I*^2^ = 15.1%). No significant differences were found comparing grade B/C DGE (25 studies), bile leak (14 studies), reoperation (26 studies), R0 (27 studies), and harvested lymph nodes (28 studies) across the three surgical approaches. The league table for all measured outcomes was shown in Table 2. SUCRA ranking is reported in Supplementary Fig. 2.

**Table 2:** League table

**LapPD**	0.81 (0.65–1.16)	0.66 (0.45–1.08)	Postoperative mortality
1.26 (0.91–1.61)	**OpenPD**	0.78 (0.54–1.12)	
1.49 (0.89–2.03)	1.23 (0.90–1.84)	**RobPD**	
**LapPD**	0.95 (0.74–1.22)	0.82 (0.58–1.13)	Grade B/C POPF
1.12 (0.82–1.43)	**OpenPD**	0.87 (0.64–1.14)	
1.21 (0.88–1.71)	1.15 (0.87–1.55)	**RobPD**	
**LapPD**	0.96 (0.72–1.26)	0.89 (0.59–1.29)	Clavien–Dindo ≥ 3
1.03 (0.80–1.46)	**OpenPD**	0.93 (0.65–1.14)	
1.12 (0.78–1.69)	1.07 (0.75–1.57)	**RobPD**	
**LapPD**	1.05 (0.72–1.38)	0.93 (0.69–1.57)	Grade B/C DGE
0.98 (0–83–1.23)	**OpenPD**	0.89 (0.72–1.16)	
1.08 (0.84–1.76)	1.12 (0.81–1.47)	**RobPD**	
**LapPD**	1.34 (1.11–2.05)	0.91 (0.68–1.35)	Surgical Site Infection
0.71 (0.59–0.83)	**OpenPD**	0.68 (0.54–0.81)	
1.08 (0.76–1.54)	1.41 (1.07–1.98)	**RobPD**	
**LapPD**	1.18 (1.09–1.75)	0.94 (0.70–1.23)	Pulmonary Complications
0.81 (0.70–0.94)	**OpenPD**	0.73 (0.64–0.86)	
1.05 (0.81–1.40)	1.29 (1.07–2.41)	**RobPD**	
**LapPD**	0.94 (0.76–1.57)	0.94 (0.66–1.34)	Bile Leak
1.07 (0.76–1.61)	**OpenPD**	0.98 (0.35–1.29)	
1.09 (0.78–1–33)	1.05 (0.79–1.51)	**RobPD**	
**LapPD**	1.23 (1.16–2.34)	0.97 (0.86–1.19)	Overall Complications
0.81 (0.74–0.93)	**OpenPD**	0.79 (0.72–0.91)	
1.03 (0.94–1.13)	1.34 (1.12–2.06)	**RobPD**	
**LapPD**	1.08 (0.86–1.43)	0.89 (0.61–1.07)	Reoperation
0.94 (0.69–1.25)	**OpenPD**	0.71 (0.49–1.09)	
1.12 (0.95–1.75)	1.22 (0.91–1.52)	**RobPD**	
**LapPD**	1.18 (1.07–1.89)	0.94 (0.65–1.71)	Hospital readmission
0.81 (0.70–0.94)	**OpenPD**	0.73 (0.64–0.86)	
1.05 (0.78–1.72)	1.12 (1.03–1.91)	**RobPD**	
**LapPD**	1.21 (0.91–1.53)	0.87 (0.53–1.29)	R0
0.83 (0.68–1.59)	**OpenPD**	1.13 (0.82–1.66)	
1.12 (0.86–1.30)	0.85 (0.67–1.36)	**RobPD**	
**LapPD**	1.06 (0.82–1.33)	0.54 (0.38–0.74)	Vascular resection
0.94 (0.75–1.21)	**OpenPD**	0.51 (0.37–0.69)	
1.84 (1.34–2.62)	1.95 (1.47–2.63)	**RobPD**	
**LapPD**	148.5 (140.5–156.5)	− 10.04 (− 23.3, 3.21)	Estimated Blood Loss
− 148.5 (− 156.5 − 140.5)	**OpenPD**	− 158.5 (− 169.2 − 147.9)	
10.04 (− 3.21, 23.3)	158.5 (147.9 − 169.2)	**RobPD**	
**LapPD**	− 1.68 (− 3.63, 0.26)	0.51 (− 3.43, 4.45)	Harvested Lymphnodes
1.68 (− 0.26, 3.64)	**OpenPD**	2.18 (− 1.23, 5.61)	
− 0.51 (− 4.45, 3.42)	− 2.18 (− 5.61, 1.23)	**RobPD**	
**LapPD**	1.92 (0.77 − 3.01)	− 0.30 (− 1.93, 1.2)	HLOS
− 1.9 (− 3.01, − 0.77)	**OpenPD**	− 2.23 (− 3.6, − 0.99)	
0.3 (− 1.2, 1.93)	2.23 (0.99 − 3.6)	**RobPD**	
**LapPD**	− 60.9 (− 67.98, − 53.9)	− 27.8 (− 38.6, − 17.1)	Operative time
60.9 (53.9–67.98)	**OpenPD**	33.1 (24.02–42.3)	
27.8 (17.1–38.6)	− 33.1 (− 42.3, − 24.0)	**RobPD**	

*OpenPD* Open Pancreaticoduodenectomy, *LapPD* Laparoscopic Pancreaticoduodenectomy, *RobPD* (obotic Pancreaticoduodenectomy. *POPF*: Postoperative pancreatic fistula; *DGE*: Delayed gastric emptying; *HLOS*: Hospital length of stay

Values are expressed as Risk Ratio (*RR*) and 95% Credible Intervals (95%CrI)

Values in each column represent the relative effect of the referral treatment (bold) with the comparator

## Discussion

This study showed that pure OpenPD, LapPD, and RobPD appears to be equally safe with comparable postoperative mortality, grade B/C POPF, and severe postoperative complications (Clavien-Dindo ≥ 3). Compared to OpenPD, LapPD and RobPD seems to be associated with significantly reduced blood loss, hospital length of stay, readmission, infectious, pulmonary, and overall complications. R0 margins and total number of harvested lymph nodes were similar across treatments.

Over the last two decades, substantial improvements have been demonstrated in the management of pancreatic head and periampullary neoplasms with an increased enthusiasm for minimally invasive approaches [71, 72]. LapPD has been shown to be comparable to Open PD in terms of safety and oncologic results [2]. However, technical limitation and poor ergonomics have made radical oncological dissection and anastomosis fashioning challenging. The advent of robotic platforms has brought new enthusiasm because of the better ergonomics and high-definition 3D visualization. Furthermore, its improved instruments motion range has led to an enhanced dissection of the uncinate process, retroportal lamina propria*,* and lymphnodes combined with an easier anastomosis fashioning [9]. Despite these technical advancements, postoperative mortality and morbidity are reported up to 3% and 30–40% in referral centers [3]. This network analysis showed that postoperative mortality was equivalent comparing OpenPD, LapPD, and RobPD with a low related heterogeneity (*I*^2^ = 18.3%), thus demonstrating a minor divergence within studies. Notably, the recent advancements of critical care have significantly improved postoperative course with reduced mortality therefore, this effect should be considered while interpreting this result. Additionally, preoperative patient selection bias, surgeon learning curve, hospital volume, and the non-specified individual-patient cause of death may be potential source of bias. Clinically relevant POPF (Grade B/C) has been shown to be a foremost contributor to major morbidity, mortality and has been reported in up to 20% of patients [73]. Independent risk factors for POPF include age, technique for anastomosis, patient comorbidities, size and consistency of the pancreatic duct, parenchyma, and low cardiopulmonary reserve [74]. We found that grade B/C POPF was comparable among the OpenPD, LapPD, and RobPD groups. This result should be interpreted cautiously because of variability in surgical techniques, postoperative prophylactic octreotide use, pancreatic duct diameter, gland texture (soft vs. firm/hard), surgical anastomotic techniques (invagination vs. duct-to-mucosa), surgeons’ experience/learning curve, and hospital volume. However, the related heterogeneity was low (*I*^2^ = 21.2%) with narrow 95%CrI, thus adding further consistency to the result. Severe postoperative complications (Clavien-Dindo ≥ 3) were found to be equivalent comparing OpenPD, LapPD and RobPD. The related global heterogeneity was low (*I*^2^ = 24.2%). However, baseline comorbidities and the heterogeneity in studies reporting may marginally influence this variability. Despite the lack of statistical significance, the SUCRA evaluation ranked RobPD as the surgical approach with the lowest probability to be ranked as first treatment for mortality (4%), POPF (13%), and postoperative Clavien-Dindo ≥ 3 (29%).

Compared to OpenPD, LapPD and RobPD were associated with a significantly reduced infectious, pulmonary, and overall complications. This is probably due to the reduced tissue trauma, postoperative pain, pulmonary impairment, and systemic stress response [2, 12]. Similarly, LapPD and RobPD were associated with significantly reduced blood loss, hospital length of stay, and hospital readmission when compared to OpenPD. The decreased blood loss and reduced transfusion requirement may presumably preserve patients’ immune system with a possibility of enhancing anti-neoplasm response [8, 12]. This is in line with the article by Kazanjian and colleagues, that reported improved survival rate following PD for pancreatic head neoplasm in patients with limited operative blood loss (< 400 ml) [75]. Moreover, the reduced SSI, pulmonary, and overall complications may allow an easier access to adjuvant treatments and potentially improve survival [76]. However, results should be interpreted cautiously because of the moderate/high related heterogeneity, probably influenced by patients’ comorbidities, preoperative patients’ selection, BMI, antibiotic therapy, ASA grade, smoke status, tumor types and size, surgical technique, need for vascular resection, and surgeon experience.

Tumor free resection margin (R0) and total number of harvested lymph nodes were similar across treatments. Again, these results need to be cautiously interpreted because of possible confounders related to different tumor size, histology, grading, presence of perineural infiltration, vascular resection, and neoadjuvant treatment. Compared to LapPD, RobPD was associated with a significantly reduced conversion rate (RR 0.71; 95%CrI 0.60–0.89; *I*^2^ = 15.1%%). This may be attributable to a more precise dissection in narrow spaces, better ergonomics, improved stability, articulated instruments manoeuvrability with highly defined 3-D anatomical dissection planes and neurovascular structures visualization [11, 12].

Opponents to minimally invasive technique may argue that longer operative times, overall increased health-care costs combined with the limited superiority do not justify the use of minimally invasive techniques. This network analysis showed statistically significant longer operative time for LapPD and RobPD, might be due to the learning curve and docking of the robot. Few articles reported the analysis of costs with a trend towards greater cost in minimally invasive techniques, mainly RobPD, probably due to the maintenance of instrument and equipment costs [77]. However, the reduced postoperative complications, hospital length of stay, and hospital readmissions may suggest a presumed ultimate cost-effectiveness [78].

LapPD and RobPD are associated with procedure-specific learning curves, however, conclusive results assessing the number of procedures required to overcome the learning curve are still inconclusive. Speicher et al. in their single high-volume center study demonstrated a significant reduction in operating time after 10 LapPD and significant reduction of blood loss after 50 procedures compared to OpenPD [34]. Sharpe et al. showed in their national database study, a comparable LapPD-related postoperative mortality after 10 operations with OpenPD [33]. Choi et al. concluded that 40 LapPD is the minimum number of procedures necessary to reach technical competence [79]. In the setting of RobPD, Boone et al. showed in their single high-volume center study, several inflection points associated with significant improvement in estimated blood loss and conversion rate (20 cases), reduction of POPF (40 cases), and improvement in operative time (80 cases) [80]. Pancreatic resection in high-volume teaching centres with adequate preoperative training programs, intraoperative coaching, and dedicated staff has been reported to be associated with significant improvements in surgical outcomes, quality of surgical resection, and survival [81]. Therefore, while the majority of included studies were performed in high-volume center, our results may not be generalized to small community hospitals.

Limitations related to exclusion of non-English written articles and heterogeneity for some of the included studies (i.e., patients’ demographics, comorbidities, inclusion/exclusion criteria, variability in operative technique, and pathological data) should be considered. Detailed cancer staging, histologic subtype, neoadjuvant treatment, type of induction therapy, and tumor location are lacking in some studies and reported as aggregated in other studies. Given that these clinical factors may impact outcomes, the lack of these components should be considered as additional confounder. In addition, patients’ treatment allocation was heterogeneous among studies and may denote a preoperative confounding and selection bias. Only three RCTs were included in the final analysis with most of the studies being low-quality non-randomized observational studies. This constitute the principal limitation of this meta-analysis and should be considered while interpreting our results. Imprecision must be considered in some of the outcomes because of the credible interval crossing the null value or include values favouring either treatment. The treatment ranking should be cautiously interpreted because it does not consider the magnitude of differences in effects between treatments and therefore chance may explain any apparent difference. Finally, no data were available on postoperative medium-/long-term survival effect of minimally invasive approaches and this mandates further investigations.

We believe that this network meta-analysis updates and broadens Ricci et al. and Kamarajah and colleagues’ studies [11, 12]. The research focus on pure surgical approaches. Therefore, articles which include hybrid hand-assisted and differently combined demolitive/reconstructive laparoscopic-robotic technique, out of the standard approaches were excluded in attempt to obtain more homogeneous data. Second, in effort to control the effect of the early learning curve and obtain more solid data, studies with less than 20 patients per arm were excluded a priori. The study was intended in accordance with PRISMA guidelines and followed a robust methodology apriori registered in the PROSPERO protocol. This generates a homogenous cohort of patients as confirmed by low heterogeneity seen in the primary outcomes.

## Conclusions

This network meta-analysis shows that the treatment of periampullary and pancreatic head tumors is evolving. Pure OpenPD, LapPD, and RobPD seems to be equivalent in terms of safety. Compared to OpenPD, both LapPD and RobPD seem associated with reduced risk of infectious, pulmonary, overall complications, blood loss, postoperative bleeding, hospital length of stay, and hospital readmission. Retrieved lymph nodes, tumor‐free resection margins, clinically relevant POPF, severe postoperative complications, and clinically relevant DGE appear to be comparable. We advocate surgeons to choose their preferred surgical approach according to their expertise however, the adoption of minimally invasive techniques may possibly improve patients’ outcomes.

## Electronic supplementary material

Below is the link to the electronic supplementary material.

Supplementary file1 (DOCX 18 KB)

Supplementary file2 (TIF 198 KB)

## Data Availability

Data generated at a central, large-scale facility, available upon request.
