# Supplementary material for: Systematic review and updated network meta-analysis comparing open, laparoscopic, and robotic pancreaticoduodenectomy
Source: Updates Surg. 2020 Dec 14;73(3):909–22. doi: 10.1007/s13304-020-00916-1 (PMC8184540; doi:10.1007/s13304-020-00916-1)
Supplement: Supplementary file 1 — Supplementary file1 (DOCX 18 KB) [file 13304_2020_916_MOESM1_ESM.docx]

| **Author** | **Confounding Bias** | **Selection Bias** | **Classification Bias** | **Intervention Bias** | **Missing Data Bias** | **Measurement Bias** | **Reporting Bias** | **Bias** |
| --- | --- | --- | --- | --- | --- | --- | --- | --- |
| Asbun et al, 2012, USA [28] | n | py | n | pn | py | pn | pn | moderate |
| Croome et al, 2014, USA [29] | py | py | n | n | n | n | py | moderate |
| Dokmak et al, 2014, France [30] | py | pn | y | y | py | py | pn | serious |
| Sharpe, 2014, USA [31] | pn | pn | pn | pn | py | n | py | moderate |
| Speicher et al, 2014, Durham, NC [32] | py | py | pn | py | py | pn | pn | serious |
| Tee et al, 2015, USA [33] | pn | py | n | py | py | n | py | moderate |
| Tran et al, 2015, USA [34] | py | py | py | py | py | py | pn | serious |
| Senthilnathan et al, 2015, India [35] | py | pn | py | pn | pn | pn | py | moderate |
| Tan, 2015, China [36] | y | py | y | pn | py | py | py | serious |
| Song et al, 2015, South Corea [37] | pn | pn | pn | py | py | pn | py | moderate |
| Kantor, 2016, USA [38] | pn | py | pn | pn | pn | py | pn | moderate |
| Delitto et al, 2016, USA [39] | py | py | py | pn | pn | pn | pn | serious |
| Stauffler et al, 2016, USA [40] | py | py | py | pn | pn | pn | n | moderate |
| Conrad et al, 2017, USA [41] | py | y | y | y | py | py | py | serious |
| Chopinet, 2017, Ftance [42] | py | py | pn | py | py | pn | pn | moderate |
| Chapman, 2018, USA [44] | pn | pn | py | pn | pn | py | py | moderate |
| Chen, 2018, China [46] | py | y | py | y | py | pn | py | serious |
| Kuesters, 2018, Germany [47] | pn | y | y | py | py | py | py | serious |
| Meng et al, 2018, China [48] | py | py | pn | y | y | pn | py | serious |
|  |  |  |  |  |  |  |  |  |
| Buchs, 2011, USA [50] | py | y | y | py | py | py | py | serious |
| Lai,2012, China [51] | y | y | py | py | pn | y | py | serious |
| Baker, 2015, USA [52] | py | pn | pn | pn | py | py | pn | moderate |
| Chen, 2015, USA [53] | pn | pn | pn | pn | py | pn | py | moderate |
| Girgis, 2016, USA [54] | py | pn | pn | pn | py | pn | pn | moderate |
| Boggi, 2016, Italy [55] | pn | pn | pn | py | pn | py | pn | moderate |
| McMillan, 2016, USA [56] | py | pn | pn | pn | py | pn | py | moderate |
| Zureikat, 2016, USA [57] | py | py | py | pn | pn | pn | py | moderate |
| Varley, 2018, USA [58] | py | py | pn | py | py | py | pn | serious |
| Kauffmann, 2018, Europe [59] | py | py | pn | pn | pn | py | pn | moderate |
| Napoli, 2018, Italy [60] | py | py | py | pn | py | py | pn | serious |
| Wang, 2018, Taiwan [61] | py | pn | pn | py | pn | py | pn | moderate |
| Cai, 2019, USA [62] | py | pn | pn | py | pn | pn | py | moderate |
| Marino et al, 2019, Italy [63] | pn | pn | pn | py | py | pn | pn | moderate |
|  |  |  |  |  |  |  |  |  |
| Liu, 2017, China [64] | py | y | py | py | py | py | pn | moderate |
| Nassour, 2017, USA [65] | py | pn | py | py | py | pn | py | moderate |
| Nassour, 2018, USA [66] | py | y | py | y | py | pn | py | serious |
|  |  |  |  |  |  |  |  |  |
| Zimmerman, 2017, USA [67] | py | py | y | pn | py | py | py | serious |
| Xourafas et al, 2018, USA [68] | pn | py | pn | pn | py | py | py | serious |

**Supplementary Table 2**. Quality assessment of the included studies (ROBINS-I tool). Each domain is evaluated with one of the following: y “yes”, py “probably yes”, pn “probably no”, and n “no”. The categories of judgement for each study are low, moderate, serious, and critical risk of bias.
