# Supplementary figures and images for: Systematic review and updated network meta-analysis comparing open, laparoscopic, and robotic pancreaticoduodenectomy
Source: Updates Surg. 2020 Dec 14;73(3):909–22. doi: 10.1007/s13304-020-00916-1 (PMC8184540; doi:10.1007/s13304-020-00916-1)

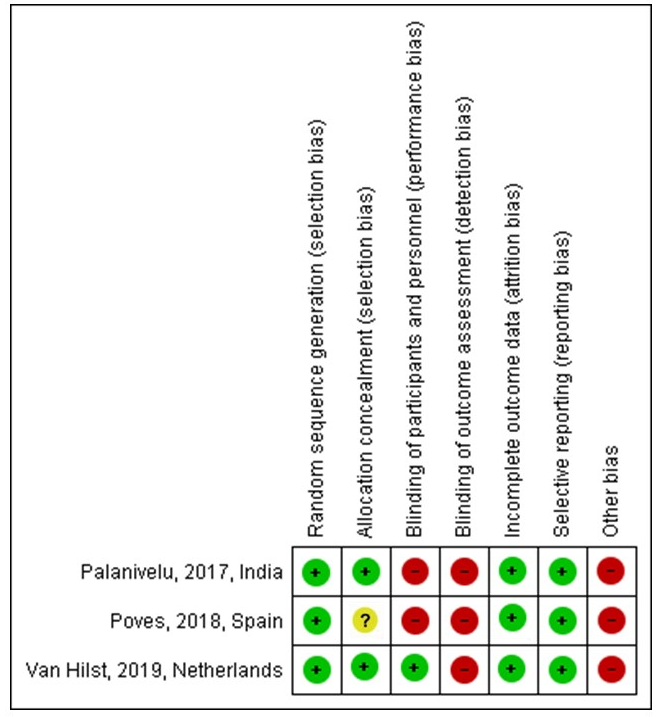

Supplement: Supplementary file 2 — Supplementary file2 (TIF 198 KB) [file 13304_2020_916_MOESM2_ESM.tif]
